# Research Progress on the Experimental Model and Underlying Mechanistic Studies of Tension-Type Headaches

**DOI:** 10.1007/s11916-024-01238-2

**Published:** 2024-03-19

**Authors:** Guo-jing Fu, liu-ding Wang, Xian-su Chi, Xiao Liang, Jing-jing Wei, Zhi-hong Huang, Wei Shen, Yun-ling Zhang

**Affiliations:** 1 Xiyuan Hospital of China Academy of Chinese Medical Sciences, Beijing, 100,091 China; 2 Yidu Central Hospital of Weifang, Weifang, 262,550 China

**Keywords:** Tension-type headache, Animal model, Pathophysiology, Adenosine triphosphate, Nerve growth factor

## Abstract

**Purpose of Review:**

Tension-type headaches (TTH) significantly diminish patients’ quality of life and increase absenteeism, thereby imposing a substantial economic burden. Animal models are essential tools for studying disease mechanisms and drug development. However, until now, little focus has been placed on summarizing the animal models of TTH and associated mechanistic studies. This narrative review discusses the current animal models of TTH and related mechanistic studies to provide insights into the pathophysiological mechanisms of and treatments for TTH.

**Recent Findings:**

The primary method for constructing an animal model of TTH involves injecting a solution of pain relievers, such as adenosine triphosphate, nerve growth factor, or a high concentration of salt solution, into the neck to initiate harmful cervical muscle responses. This model enables the examination of the interaction between peripheral muscles and central sensitization, which is crucial for understanding the pathophysiology of TTH. Mechanistic studies based on this model have investigated the effect of the P2X receptor antagonist, P2X7 receptor blockade, the P2Y1 receptor agonist 2-MESADP, P2Y1 receptor antagonist MRS2179, nitric oxide synthase inhibitors, and acetylsalicylic acid.

**Summary:**

Despite notable advancements, the current model of TTH has limitations, including surgical complexity and the inability to replicate chronic tension-type headache (CTTH). To gain a more comprehensive understanding and develop more effective treatment methods, future studies should focus on simplifying surgical procedures, examining other predisposing factors, and establishing a model for chronic TTH. This will offer a deeper insight into the pathophysiological mechanism of TTH and pave the way for improved treatment approaches.

## Introduction

Tension-type headache (TTH) is a widely prevalent primary bilateral headache of mild to moderate intensity characterized by a pressing or tightening sensation and not aggravated by routine physical activity. Depending on the frequency, TTH can be subdivided into infrequent episodic tension-type headaches (IFETTH), frequent episodic tension-type headaches (FETTH), and chronic tension-type headaches (CTTH). Data from the Global Burden of Diseases, Injuries, and Risk Factors Study 2016 (GBD 2016) revealed that approximately 3 billion individuals globally suffer from headaches, of which 1.89 billion experience TTH. Upon investigating 328 diseases, GBD 2016 identified TTH as the third most prevalent condition, following dental caries and latent tuberculosis infection. In 2016, TTH accounted for 7.2 million years lived with disability (YLDs), marking a 5.31% increase from 1990 [1•]. This ailment significantly impairs the productivity and quality of life of patients, thus posing an economic burden worldwide [1•, 2, 3]. Furthermore, TTH has been shown to considerably affect mood and sleep, with anxiety or depression being more common in patients with TTH than in unaffected individuals [4–6].

The pathophysiology of TTH primarily encompasses peripheral and central factors. Muscle factors play a pivotal role in TTH progression, with patients generally exhibiting increased pericranial muscle tenderness [7, 8], especially neck pain [9]. Tenderness of the trapezius muscle is commonly reported [7, 10, 11]. Chronically stressed or anxious individuals hunch their shoulders, creating tension in their shoulders and neck, which constantly strains the trapezius. Despite the inconsistent data, pericranial muscle tenderness may correlate with headache intensity, frequency, and duration [9, 10, 12]. Pericranial muscle tenderness may be a critical risk factor for headache development; however, whether muscle tenderness is a consequence of pain or the cause of a TTH attack remains disputable [13•, 14]. Myofascial trigger points (MTrPs), which are hyperirritable spots linked with a taut band of a skeletal muscle that typically responds with a referred pain pattern far from the spot, may play a critical role in the pathophysiological mechanism of TTH [15]. The formation of MTrPs involves several factors, such as muscle overuse, mechanical overload, or psychological stress. Once formed, they reflexively interfere with normal muscle function through different pathways, either causing muscle tension and sustained contraction or excessively compressing small blood vessels, leading to hypoxia, ischemia, and producing painful metabolites.

Consequently, this can increase the sensation of myogenic injury [16, 17]. Various factors related to pain and inflammation, such as bradykinin, calcitonin gene-related peptide (CGRP), and substance P (SP), have been found in active MTrPs [18, 19]. Numerous studies have confirmed that the number of MTrPs in patients with TTH exceeds that in healthy individuals [20–22]. MTrPs are found predominantly in the head and neck muscles of patients with TTH, such as temporalis, suboccipital, sternocleidomastoid, and upper trapezius muscles. They can cause referred pain similar to TTH when compressed and stretched. Although current studies have demonstrated that MTrPs in the head and neck muscles are associated with TTH, there is a lack of longitudinal studies evaluating the role of MTrPs in the development of TTH, hence failing to establish a causal relationship between MTrPs and CTTH.

Acidic tissue pH is one of the primary activators of peripheral muscle pain [23]. Several stimuli, such as MTrPs, chronic ischemic states, muscle overuse, and tonic contractions or spasms, are accompanied by a drop in tissue pH. These stimuli activate purinergic and vanilloid receptors, inducing the release of neuropeptides (SP and CGRP) and a variety of endogenous factors (bradykinin and E2-prostaglandins), triggering the activation of thin myelinated (Aδ) and unmyelinated (C) fibers of the myofascial nociception, and leading to the sensitization of peripheral nerves, such as the trigeminal and greater occipital nerves [13•, 14, 24].

Central sensitization could be critical in the transformation of FETTH into CTTH. Persistent nociceptive input from myofascial structures, that is, peripheral sensitization of muscle nociceptors due to releasing an algogenic factor, may induce central sensitization in cases of CTTH [15]. This central sensitization is attributed to alterations in the neuronal properties of the central nervous system [25]. Sensitization of second-order neurons at the spinal dorsal horn/trigeminal nucleus level, sensitization of supraspinal neurons, and reduced antinociceptive activity from supraspinal structures may also contribute to TTH chronification [13•, 15]. Despite identifying several cellular pathways involved in central sensitization, the underlying mechanism of CTTH remains unclear [26].

Noxious stimulation or peripheral inflammation could release neurotransmitters such as glutamate, SP, and CGRP in the spinal dorsal horn. These neurotransmitters can activate receptors such as NMDAR, mGluR, NK1R, B2, and CGRP1, which release intracellular Ca^2+^, which then activates protein kinase A (PKA), protein kinase C (PKC), calmodulin dependent protein kinase II (CaMKII), and extracellular regulated protein kinases (ERK). This series of biochemical events, including an increase in nitric oxide (NO), prostaglandins, and protein kinases, plays a crucial role in maintaining the central sensitization of spinal neurons [25, 27].

The prevalence of TTH exhibits a significant sex difference, with higher rates in woman than in men [1•]. This sex difference may, to some extent, facilitate our understanding of the mechanisms behind TTH. There are several possible reasons for this sex difference. (a) There is a sex difference in pain perception, which may be related to the regulation of pain by hormones as well as differences in brain structural development and functionality (such as the volume of different cortical areas associated with pain) [28, 29]. (b) Psychological, emotional, and social sex differences may be associated with the maintenance of chronic pain. For example, women are more prone to depression and anxiety disorders than men, and they demonstrate more pronounced expressions of pain [29]. (c) There are sex differences in nociceptive muscle pain: the number of TrPs is higher in women with TTH than in men with TTH, and the associations between muscle pain, anxiety, and stress nociceptive sensitization is more pronounced in women than in men [30]. (d) There are sex differences in emotional triggers: the headache burden in men with TTH was correlated with sleep quality, whereas the headache burden in women with TTH was most strongly correlated with depression levels and headache intensity [31, 32].

**Fig. 1:**
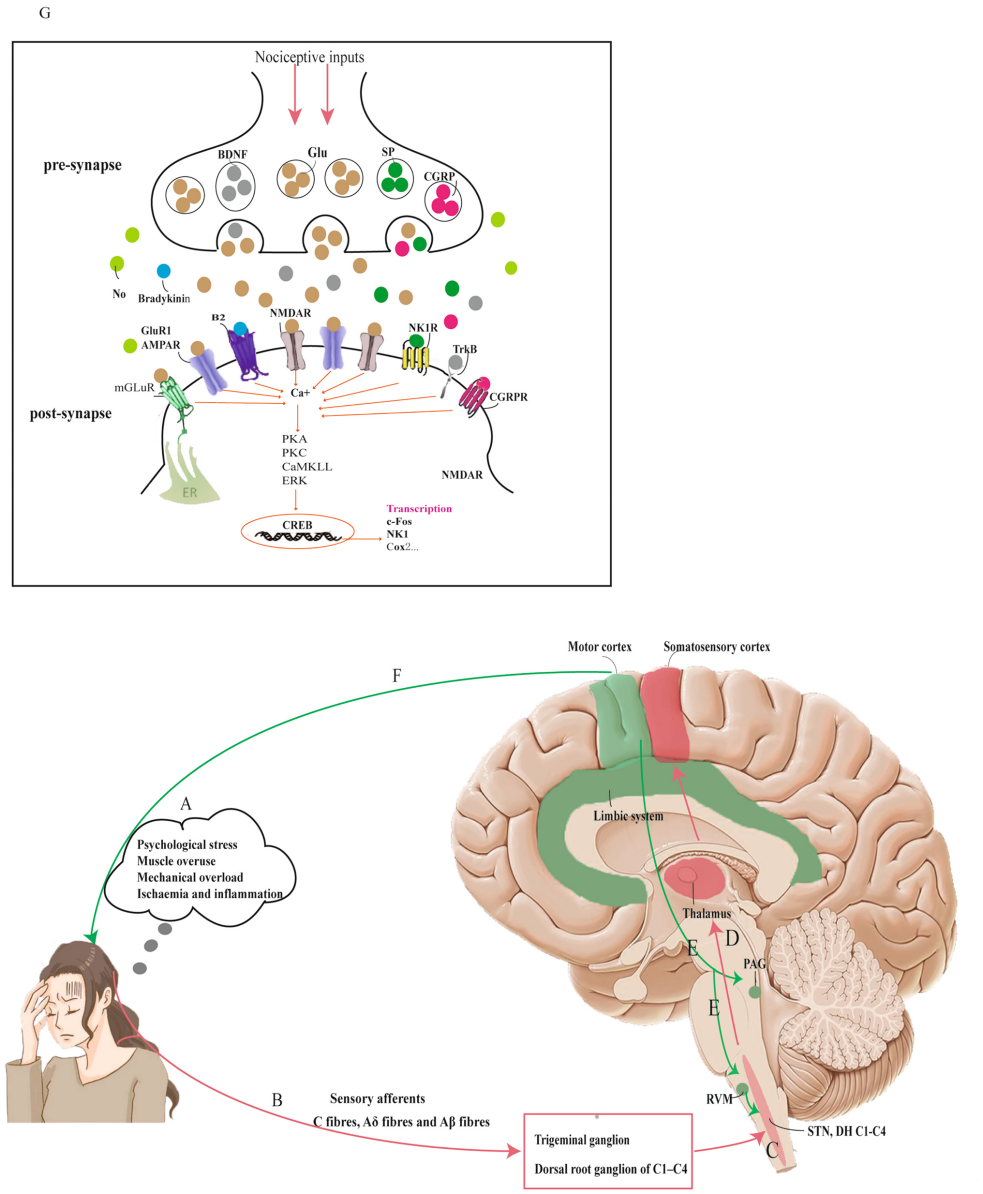
Pathophysiological model of tension-type headache (TTH). **A** Muscle overuse, mechanical overload, psychological stress, ischemia, and inflammation, along with the release of various chemical mediators due to local pathological conditions, activate and sensitize muscle nociceptors, and peripheral sensory afferent fibers. **B** The trigeminal nerve and C1–C4 dorsal root ganglia receive nociceptive information from the periphery via C fibers, Aδ fibers, and Aβ fibers. **C** Increased peripheral noxious input leads to the sensitization of secondary neurons in the spinal trigeminal nucleus and dorsal horns at the C3–C4 level. **D** The heightened nociceptive input from the spinal trigeminal nucleus and dorsal horns results in the sensitization of supraspinal neurons. Pathways A, B, C, and D (highlighted in red) illustrate the regulatory process of ascending pain conduction. **E** Enhanced nociceptive activation of supraspinal structures may result in increased facilitation (via RVM) and decreased inhibition (via PAG) of pain transmission at the level of the spinal dorsal horn/trigeminal nucleus. **F** The heightened nociceptive activation of supraspinal structures enables descending inputs from the motor cortex to regulate motor neurons. This regulation leads to increased pericranial muscle activity, which manifests as increased muscle contraction or heightened muscle rigidity. Pathways E and F (highlighted in green) depict the control process of pain perception through descending input, triggered by increased stimulation of spinal cord structures. **G** Schematic representation of the molecular mechanisms underlying central sensitization [35]. This process mainly pertains to the sensitization of second-order neurons in the spinal dorsal horn/trigeminal nucleus, as well as sensitization of supraspinal structures. Neurotransmitters such as substance P, glutamic acid, and procalcitonin may contribute to the development of central sensitization. Normally dormant postsynaptic receptors, including NMDA receptors, mGluR receptors, and NK1R receptors, may be activated by the release of these neurotransmitters. The activation of these receptors induces an increase in calcium influx, which in turn triggers PKA, PKC, CaMKII, and ERK. This catalyzes a series of biochemical processes, including the increase of nitric oxide, prostaglandins, protein kinases, and immediate early gene protein products

Adenosine phosphate (ATP), an essential regulator of injurious processes, can augment glutamate release and enhance central sensitization through its presynaptic release via the central terminal. This process forms the foundation of many chronic pain disorders [33]. NO, a key transmitter in spinal cord pain pathways, contributes to establishing and maintaining the central sensitization [34]. A model of the pathophysiology of TTH is illustrated in Fig. 1.

In this narrative review, we discuss the current animal models of TTH and related mechanistic studies to provide insights into the physiopathological mechanism and treatment of TTH. We also summarize the limitations of existing research.

## Theory and Method of Establishing an Animal Model of TTH

The role of neck muscle nociception in the pathogenesis of TTH is crucial, likely influencing the persistent facilitation of brainstem nociception [36•]. Given this pathophysiology, various animal models have been developed to investigate the impact of nociceptive afferent input from neck muscles on processing within the central nervous system [36•, 37–42]. ATP and nerve growth factor (NGF) are predominantly used to create animal models of muscle pain [23]. The primary methodology for creating a TTH animal model involves neck injections of an algesic solution containing hypertonic saline, NGF, or ATP [43]. A nociceptive solution stimulates nociceptive neck muscle afferents, consistently promoting brainstem nociception in model animals.

Several studies have successfully established TTH animal models by injecting intramuscular algesic solvents directly into the neck muscles. In particular, following anesthesia, an injection cannula (approximately 0.40 mm in diameter) is inserted into the bellies of both semispinalis neck muscles. Glass microsyringes are connected to a liquid switch via thin tubing and secured to a microdialysis pump. Injecting ATP, NGF, or hypertonic saline into both semispinalis neck muscles mimics the bilateral neck muscle pain experienced by patients with TTH, thus effectively establishing a TTH animal model [36•, 37–40, 42].

The jaw-opening reflex (JOR), triggered by electrical stimulation of afferents in the tongue muscle, is primarily utilized to ascertain the successful generation of model animals. The reflex response in the anterior belly of the digastric muscle is recorded using electromyographic activity (EMG). The spinal trigeminal nucleus, receiving convergent inputs from primary sensory fibers (neck muscle, tongue), projects onto the digastric motoneurons [44]. Consequently, additional excitatory input from neck muscle nociceptors facilitates heterosynaptic access to the reflex neural network [38]. Nöbel et al. [45] established a TTH model by injecting α,β-methyleneadenosine 5′-triphosphate (α,β-meATP) into the ipsilateral temporal muscle of mice. This heightened the continuous activity of spinal trigeminal neurons receiving afferent input from the temporal muscle and cranial dura mater.

## Studies on Establishing An Animal Model of TTH with ATP

Among the three algesic compounds, ATP is the most potent in inducing pain input from neck muscles, indicating it is an appropriate chemical stimulant. Following cellular injury, ATP is released into the extracellular medium in millimolar quantities, driving inflammatory and nociceptive processes through the stimulation or sensitization of peripheral nociceptors [46, 47]. Likewise, muscle contraction triggers a surge in the interstitial concentration of ATP [48–50]. In healthy individuals, intramuscular ATP injection induces moderate to severe pain and tenderness [51]. ATP plays a significant role in excitatory injury responses via P2X (P2X1-7) and P2Y receptors [37, 52–55]. In vivo pain and inflammation models, created by injections of ATP and α,β-meATP into rats’ hind paws and knee joints, have demonstrated the activation of P2X receptors on sensory neurons [55–57]. Owing to the short half-life of ATP, the ATP analog α,β-meATP is often used to generate experimental animal models. Compared with ATP, this analog offers enhanced metabolic stability and sensitivity to P2X1, P2X2, and P2X2/3 receptors [50].

Numerous studies have established TTH animal models by injecting α,β-meATP intramuscularly into the neck. These studies have focused mainly on conducting mechanistic studies, including delineating the effect of P2X and P2X7 receptor blockade, P2Y1 receptor agonists and antagonists, NO synthase inhibitors, and acetylsalicylic acid. In this review, we discuss all these relevant studies.

### P2X Receptor Antagonist, PPADS

Pyridoxalphosphate-6-azophenyl-2′,4′-disulphonic acid (PPADS) is a broad-selectivity P2 receptor antagonist, with P2X1, P2X2, P2X3, and P2X5 receptors being most sensitive [58–60]. Studies have indicated that PPADS can diminish the pain sensation in a mouse model of neuropathic pain, effectively reducing both tactile allodynia and thermal hyperalgesia in a dose- and time-dependent manner [60, 61]. For instance, 25 mg/kg of PPADS fully reversed pain hypersensitivity in a neuropathic pain animal model [60, 61]. In one study, injection of 20 µL of α,β-meATP (ATP; 100 nmol/L and 1 µmol/L) into the bilateral semispinalis muscles of the neck effectively simulated the bilateral neck muscle pain characteristic of TTH [40]. The authors noted that ATP consistently promoted JOR in a dose-dependent manner for at least 2 h, confirming successful model construction. To investigate the effect of ATP on P2X receptors, PPADS (3, 10, 30, or 100 nmol/L) was administered intramuscularly 20 min prior and 35 min after ATP injection. Alterations in JOR were consistently monitored within 1 h after administration. Accordingly, pretreatment with PPADS inhibited the reflex in a dose-dependent manner, whereas PPADS posttreatment (100 nmol/L) completely restored the facilitated reflex to baseline values. Thus, the P2X receptor antagonist PPADS inhibited reflex induction dose-dependently and contributed to reflex maintenance. These findings suggest that ATP induces a lasting enhancement of craniofacial nociception via extended activation of P2X receptors in the neck muscles.

### P2X7 Receptor Blockade Using A-438079

P2X7 receptors are pivotal in neuropathic pain and are implicated in the peripheral and central regulation of the release of the interleukin-1β(IL-1β) proinflammatory cytokine [62–65]. Studies have found that the number of P2X7 receptors is elevated in damaged neural tissues of patients with chronic neuropathic pain [65]. P2X7-deficient mice demonstrated decreased inflammatory thermal hyperalgesia and mechanical allodynia generated by nerve damage [63, 65, 66]. A-438079, a P2X7 antagonist, functions as an anti-injury agent by blocking the P2X7 receptor and curtailing the release of ATP-associated IL-1β. A-438079 (10–300 mol/kg) exerted an anti-allodynic effect and alleviated formalin-induced pain in three animal models of neuropathic pain [67]. A TTH mouse model was established by injecting 20 mL α,β-meATP (intramuscularly, 20 mL/min) into the semispinal neck muscles of mice [37]. Purinergic reflex stimulation was observed for the next 90 min. Mice were then divided into four groups based on the dosage and route of administration of the antagonist: intraperitoneal injections of 150 µmol/kg and 300 µmol/kg A-438079, intramuscular injection of 100 µM A-438079, and intraperitoneal injection of normal saline. As expected, α,β-meATP reliably facilitate JOR. Conversely, intraperitoneal injection of A-438079 (150 µmol/kg and 300 µmol/kg) fully reversed the effects of α,β-meATP in a dose-dependent manner. However, brainstem nociceptive processing was unaffected by saline or intramuscularly administered A-438079 (100 µM). Multiple studies have shown widespread expression of P2X7 in neuroglia of the central nervous system [68–70], where microglial P2X7 receptors appear to be involved in long-term potentiation (LTP) of spinal nociceptive information processing [62, 71]. Electrical stimulation of the dorsal root ganglion can also lead to ATP release, inducing the pain process and facilitating neuron–glia signaling [70, 72]. This study suggested that central nervous system P2X7 receptors facilitate the brainstem nociceptive processing of neck muscles. As the effects of A-438079 mimic the acute treatment of TTH, the P2X7 receptor may serve as a future therapeutic target for TTH management.

### P2Y1 Receptor Antagonist and Agonist, MRS2179 and 2-MESADP

P2Y receptors are metabotropic receptors that transduce ATP neurotransmitter signals by activating various intracellular signal transduction pathways via G proteins [73, 74]. ATP interacts with both ionotropic P2X receptors and metabotropic P2Y receptors [74]. Among them, the P2Y1 receptor was reported to inhibit excitability by mediating the conductance of P2X3 receptor channels [75–77].

To verify the inhibitory effect of P2Y1 on ATP-induced neck muscle nociception, a three-part experiment was conducted [50]. In the initial part, the authors observed the changes in JOR for at least 2 h following the intramuscular injection of α,β-meATP (100 nmol/L, 1 µmol/L) or ATP (100 nmol/L, 1 µmol/L, 7.6 mmol). The second phase involved pretreatment with the competitive P2Y1 receptor antagonist 2′-deoxy-*N*^6^-methyladenosine 3′,5′-bisphosphate tetrasodium salt (MRS2179 1 µmol/L) followed by the intramuscular injection of ATP (1 µmol/L). JOR was observed for 2 h after treatment. The third part consisted of the intramuscular injection of α,β-meATP (1 µmol/L) in the neck and JOR monitoring for 2 h, followed by the injection of the P2Y1 receptor agonist 2-(methylthio)adenosine 5′-diphosphate trisodium salt hydrate (2-MeSADP 1 µmol l/L) or saline and monitoring of the reflex effect for another 2 h. This study demonstrated that reflex facilitation induced by natural ATP occurred only at the lowest concentrations (100 nmol/L). ATP exhibits a high affinity for P2X receptors; at higher concentrations, it also binds to P2Y receptors. Conversely, its primary metabolite, ADP, shows a stronger affinity for P2Y1 receptors [78, 79]. Notably, ATP’s excitatory effect on P2X receptors is predominant at the low 100 nmol/L concentration. However, at higher concentrations of natural ATP, the concurrent binding of ADP to inhibitory P2Y1 receptors may counteract ATP’s influence on excitatory P2X receptors. This study confirmed that pretreatment with the competitive P2Y1 receptor antagonist MRS2179 induced reflex facilitation even at high ATP doses, revealing the inhibitory effect of P2Y1 receptors on the excitatory effects of elevated ATP concentrations and metabolites. Conversely, the P2Y1 receptor agonist 2-MeSADP efficiently reversed established reflexes mediated by the α,β-meATP-established reflex back to the baseline within an hour, indicating complete reversal by P2Y1 receptors. This study emphasizes the significance of distinct interactions between purinergic P2X and P2Y1 receptors in developing future pharmaceutical treatments for TTH of craniofacial nociception by extended activation of P2X receptors in the neck muscles.

### NOS Inhibitors

Nitric oxide synthase (NOS) catalyzes NO formation, facilitating signal transmission in the central and peripheral nervous systems [80, 81]. Types of NOS include neuronal NOS (nNOS), inducible NOS (iNOS), and endothelial NOS (eNOS). Animal studies have suggested that nNOS and NO production stimulation could be associated with central sensitization [80, 82]. Therefore, inhibiting NOS may decrease central sensitization in animal models of chronic pain [83–85].

Research on NOS inhibitors, such as NG-monomethyl-l-arginine acetate (L-NMMA), Nω-propyl-l-arginine (NPLA), and 1400W, is a hot topic. One study demonstrated that the nNOS inhibitor L-NMMA reduced headache intensity and pericranial muscle stiffness and tenderness in patients with chronic TTH [80]. Hence, NOS inhibition might be an effective approach for treating CTTH in the future [39, 80, 83].

Two studies investigated NOS inhibitors’ preventative and reversal effects on ATP-induced nociceptive afferents from neck muscles [38, 39]. In particular, injection of the nonspecific NOS inhibitor L-NMMA before intramuscular administration of α,β-meATP inhibited brainstem reflex facilitation in a dose-dependent manner. Subsequent intraperitoneal injection of L-NMMA reversed this reflex facilitation to baseline levels [38]. Likewise, the nNOS inhibitor NPLA inhibited α,β-meATP-induced neck muscle nociception in anesthetized mice in a dose-dependent manner. Subsequent iNOS inhibition by 1400 W partially reversed this effect, while a subsequent injection of the nNOS inhibitor NPLA did not have any effect [39]. These findings emphasized the involvement of NOS isoenzymes in the pathophysiology of TTH and suggested that NOS inhibitors may potentially be used for treating TTH in the future [80, 86, 87].

### Acetylsalicylic Acid

Acetylsalicylic acid (ASA) is currently recognized as an acute-acting drug recommended for patients with TTH (level of recommendation, A) [88–90]. Its effectiveness has been confirmed in numerous clinical studies [91–93]. ASA’s peripheral and central analgesic effects have also been validated in various animal pain models [94–97]. Most of its therapeutic actions are suggested to derive from the inhibition of prostaglandin synthesis, mediated by cyclooxygenase (COX) [93, 98].

Ristic et al. [99] conducted two experiments on mice. The first experiment centered on enhancing the α,β-meATP-induced reflex by subsequent administration of ASA. They first constructed a mouse model of TTH by injecting α-β-meATP into the cervical semispinalis muscle of mice. After monitoring JOR for 60 min postinjection, either saline or ASA was administered intraperitoneally at doses of 15, 30, and 60 mg/kg. The response was then observed for an additional 90 min. The second experiment focused on pretreatment with ASA or saline to enhance the α-β-meATP-induced reflex. To this end, following administration of 60 mg/kg ASA, the reflex was observed for 60 min, whereas after injection of normal saline, a 30-min observation was performed. Subsequently, mice were subjected to intramuscular infusion of α,β-meATP, and JOR was monitored for another 60 min.

Compared with low doses (15 mg/kg) and saline, 30 and 60 mg/kg ASA doses reversed purinergic facilitation. In addition, pretreatment with ASA (60 mg/kg) prevented purine promotion compared with saline pretreatment. This indicated that ASA inhibits the α,β-meATP-induced enhancement of neck muscle nociception in anesthetized mice. Both pre- and post-treatment with ASA inhibited purinergic facilitation. However, the molecular target of ASA in the central nervous system against nociception remains unclear; it is hypothesized that ASA may act through COX or NOS.

## Study on Establishing an Animal Model of TTH with NGF

NGF is a potential candidate for inducing selective stimulation of group IV nociceptors in skeletal muscle through tyrosine kinase A (trkA) and p75 receptors [51, 100]. For instance, injecting NGF into the masseter muscles of healthy volunteers resulted in persistent mechanical allodynia and hyperalgesia [101], suggesting the potential role of NGF as a chemical stimulus for selective nociceptive input from individual muscles [41].

This potential was explored in a study in which they injected NGF into the unilateral and bilateral semispinalis muscles of the neck in anesthetized mice, creating a nociceptive model that demonstrated intense and sustained brainstem somatosensory processing via JOR [41]. Notably, local injection of NGF into the semispinalis neck muscles led to significant Fos immunoreactivity in the periaqueductal gray matter (PAG), lateral reticular nucleus (LRN), and superficial layers of the C1–C3 cervical spinal dorsal horn levels [102]. The neuronal activation pattern following local NGF administration was shown to align with the anatomy of the central nervous system, processing deep nociceptive inputs. Thus, this model may be suitable for studying nociception in neck muscles and may provide a future animal model of TTH.

ATP and NGF are algogenic factors frequently observed in studies concerning myofascial pain [46, 51, 101]. Although the facilitative effects of NGF and ATP are remarkably similar when intramuscularly injected, the underlying molecular pathways differ. One study investigated the molecular mechanisms of NGF and ATP by observing the impact of tetrodotoxin (TTX) on NGF- and ATP-induced myofascial nociception [36•]. They showed that pretreatment with TTX modulated ATP-induced reflexes in a dose-dependent manner. In particular, administration of TTX at 50 and 100 nmol/L concentrations before the ATP injection prevented ATP-induced brain stem sensory enhancement. Moreover, TTX administration 35 min after the ATP injection prevented additional reflex facilitation, reducing the enhanced reflex to baseline values within 1 h. However, no significant effect was observed on NGF. TTX efficiently blocks sodium currents by binding to voltage-gated sodium channels (Nav) 1.1, 1.2, 1.3, 1.6, and 1.7, preventing the generation and propagation of action potentials in sensory neurons. In contrast, Nav1.8 and 1.9 exhibit resistance to TTX inhibition [103, 104]. These different effects of TTX on ATP- and NGF-induced myofascial nociception may be due to the fact that TTX can block ATP-mediated excitatory effects on injury receptors via P2X3 and affect afferent fiber-mediated injury responses. The excitatory effect of NGF on injury receptors involves sodium currents that are resistant to TTX [105].

### Nitric Oxide Synthase Inhibitors

The influence of NOS inhibitors, particularly the selective inhibitors of nNOS, in NGF-induced TTH models, is of great importance. In one specific study investigating the effects of selective nNOS inhibition on NGF-induced reflex facilitation, the experimental design was divided into two parts [106]. Initially, the NOS inhibitor, NPLA, was administered in intraperitoneal doses of 100 µL at concentrations of 0.096 mg/kg, 0.96 mg/kg, and 1.92 mg/kg. An isotonic saline solution (100 µL, 0.9%) was administered as a control. Following this, three reflex series were recorded, and then NGF at a concentration of 0.8 µM was injected intramuscularly. Subsequent observations of the effect on JOR were conducted for 1 h.

In the second part of the study, after recording the three reflex series following the intramuscular injection of NGF, an intraperitoneal injection of NPLA at a concentration of 0.96 mg/kg was administered. The effects on reflex promotion were monitored for the subsequent 30 min.

The authors found that NPLA prevented central facilitation by painful myofascial input from the neck in a dose-dependent manner. Pretreatment with NPLA at doses of 0.96 mg/kg and 1.92 mg/kg successfully hindered the NGF-induced reflex facilitation. However, subsequent administration of NPLA at 0.96 mg/kg did not reverse the already established NGF-induced reflex promotion. Consequently, this study suggested that nNOS-selective inhibitors hold promise as potential pharmaceutical targets for treating TTH.

## Establishing an Animal Model of TTH with Hypertonic Saline

Besides ATP and NGF, hypertonic saline has been employed as an initial approach to induce a TTH model. Intramuscular injection of hypertonic saline can induce muscle pain with local and referred pain characteristics [107, 108]. Animal studies have shown that injecting a 5% hypertonic saline (20 µL) into the neck muscles promoted JOR for at least 1 h [109]. Thus, a cervical injection of hypertonic saline can simulate the pain state of TTH, suggesting that harmful cervical muscle input induced by hypertonic saline solution may cause long-term enhancement of the sensorimotor jaw reflex and pave the way for future animal models of TTH. The animal models of TTH and the corresponding mechanisms of construction are summarized in Table 1.

**Table 1:** Summary of the TTH animal model and underlying mechanistic studies

**Algesic solvents**	**Muscle**	**Drug**	**Main funding**
ATP	Both semispinalis neck muscles	P2X receptor antagonist—PPADS	These findings suggest that ATP induces a lasting enhancement of craniofacial nociception via extended activation of P2X receptors in the neck muscles [40]
ATP	Both semispinalis neck muscles	P2X7 receptor blockade—A-438079	This study suggests that central nervous system P2X7 receptors facilitate the brainstem nociceptive processing of neck muscles. As the effects of A-438079 mimic the acute treatment of TTH, the P2X7 receptor may potentially serve as a future therapeutic target for the management of TTH [37]
ATP	Both semispinalis neck muscles	P2Y1 receptor antagonist and agonist—MRS2179 and 2-MESADP	This study suggests the importance of divergent interactions between purinergic P2X and P2Y1 receptors for future pharmaceutical treatments for TTH [50]
ATP	Both semispinalis neck muscles	NOS inhibitors	These findings emphasize the involvement of NOS isoenzymes in the pathophysiology of TTH and suggest that NOS inhibitors may potentially be used for treating TTH in the future [85–87]
ATP	Both semispinalis neck muscles	Acetylsalicylic acid	Both pre- and post-treatment with ASA inhibited purinergic facilitation. However, the molecular target of ASA in the central nervous system against nociception remains unclear; it is hypothesized that ASA may act through COX or NOS [98]
NGF	Both semispinalis neck muscles	NOS inhibitors	This study suggests that nNOS-selective inhibitors hold promise as potential pharmaceutical targets for treating TTH [106]
Hypertonic saline	Both semispinalis neck muscles	——	This study suggests that harmful cervical muscle input induced by hypertonic saline may cause long-term enhancement of the sensorimotor jaw reflex and pave the way for future animal models of TTH [109]

## Current Study Limitations

Based on the literature, the current animal models of TTH lack maturity. The following limitations demonstrate this:

1. Current models of TTH mainly rely on JOR for validation. In these models, JOR has been used to demonstrate the involvement of the trigeminal nerve, primarily of the V3 or third branch, which is not as strongly implicated in headache pathology. As headache is the main symptom, a primary measure of TTH should be the study of trigeminal nociception; however, this is not typically performed.
2. Implementing animal models of TTH is intricate, with a low success rate. A team led by Hu Zhiqiang in China introduced a simplified surgical design for constructing an animal model of TTH, which was then utilized for investigating the effects and mechanism of action of traditional Chinese medicine. However, this design has not been extensively adopted [110–112]. Furthermore, the entire testing procedure is conducted under anesthesia, which inhibits the detection of behavioral indications and limits data collection. Hence, the current animal models of TTH are not ideally suited for assessing drug efficacy.
3. Applying algesic compounds in these models tends to involve single, nonrepetitive administrations. This results in animal models more closely simulating acute rather than chronic headache paradigms. This occurs despite the potential shift in pathophysiological mechanisms from episodic to chronic TTH. Consequently, the current models may be more suitable for developing drugs to treat paroxysmal TTH, whereas their utility for studying chronic TTH is limited [99]. Future studies should examine whether repeated injections of an algesic compound could construct an animal model of chronic TTH; however, this may be more difficult to achieve given the complexity of current models.
4. While numerous studies have explored the development of medications based on current animal models of TTH, most have focused on the target receptors of algesic compounds. Consequently, drug development targeting other receptors has been somewhat neglected.

Regarding the design methodology of animal models of TTH, the following aspects can be considered:

1. TTH can be triggered by many factors, including psychological elements (such as stress, anxiety, and depression), sleep disturbances, head, neck, and shoulder posture issues, and temporomandibular joint dysfunction [20, 113–115]. However, none of the current animal models of TTH accounts for these variables and should be accounted for in the future.
2. Research on migraine models has advanced considerably [116–118]. Given the similarities between migraine and TTH regarding clinical manifestations, predisposing factors, and epidemiological characteristics, distinguishing between the two can be challenging, particularly when both conditions become chronic or often co-occur [119]. Exploring the possibility of designing animal models of TTH based on established migraine models may be a fruitful avenue for future research.
3. Fibromyalgia (FM) is an important comorbidity of TTH. Many similarities can be found between the two disorders. For example, FM can manifest with clinical symptoms such as neck and occipital muscle pressure pain, often accompanied by anxiety, depression, and poor sleep. In addition, central sensitization is a mechanism shared by both disorders [13•, 120, 121]. FM’s current experimental animal models are advanced and established through various methods, such as the reserpine-, acid saline-, and stress-induced FM models. Notably, the acid saline-induced model is constructed by unilaterally injecting acidic saline (pH 4.0) twice into the gastrocnemius muscle of mice, causing sensory changes such as mechanical nociceptive hypersensitivity and abnormal mechanical muscle pain, which are accompanied by comorbidities such as depression, anxiety, and fatigue [122]. This model allows an in-depth exploration of the intrinsic mechanisms of fibromyalgia nociceptive hypersensitivity and central sensitization. The pH of acidic tissues is one of the primary activators of peripheral nociceptors and can induce muscle pain; so, whether this model can be applied for studying tension-type headache, which can be induced by injecting acid saline into the neck muscles, is a direction that can be attempted in future studies [120].
4. Given the critical role of MTrPs in the pathophysiology of TTH, a valid question would be whether animal models of MTrPs can be applied to the TTH model. Currently, MTrP models have been mainly constructed by striking the quadriceps muscle of rats combined with centrifugal movement and subcutaneous injection of cholinesterase inhibitors [123, 124]. The problem is that the generated model may be presented with a variety of diseases, including migraine, temporomandibular joint (TMJ) dysfunction, sinusitis, and cervical neuralgia, as well as a variety of otological problems, including tinnitus, otalgia, and dizziness [125]. Moreover, striking the muscles of the head and neck is not feasible. Thus, although applying the MTrPs modeling for studying TTH may be difficult, it can be used as the basis for the construction of improved models of TTH.

Considering the current animal models of TTH lack maturity, various studies of tension-type headache have been conducted based on human volunteers. Future research could draw on alternatives to animal model studies. Promising technologies to replace animal experiments include tissue bioprinting, organoids, and organ chips [126–128]. By bioprinting, neuronal constructs can more accurately replicate the microenvironment of neural tissue, which is currently an important model for understanding disease processes and developing new therapies [126]. Microphysiological neural systems on a chip can simulate various high-level physiological and pathophysiological phenotypes of the human nervous system and have been successfully applied in basic biomedical research, clinical applications, and personalized medicine for neurological diseases such as Alzheimer’s disease, Parkinson’s disease, and traumatic brain or nerve injuries [128–130]. Although these emerging technologies are still relatively lacking in research on tension-type headaches, they can provide research ideas for studying tension-type headaches.

## Conclusions

The current methodology for constructing an animal model of TTH primarily involves injecting an algesic solution into the neck, which induces noxious cervical muscle afferents. This consistently promotes nociceptive sensation within the brainstem of the animal. This model allows for investigating the crucial interplay between peripheral myofascial factors and central sensitization, which are key to TTH pathophysiology.

Accordingly, mechanistic studies, such as those involving the P2X receptor antagonist PPADS, P2X7 receptor blocker A-438079, P2Y1 receptor agonist 2-MESADP, P2Y1 receptor antagonist MRS2179, NOS inhibitors, and acetylsalicylic acid, have been conducted based on this model.

To gain a more comprehensive understanding of TTH pathophysiology, future studies employing animal models of TTH should aim to reduce operational complexity, develop a chronic headache model, and consider a broader range of predisposing factors.

## Data Availability

Details of this review are available from the corresponding author upon request.
